# A New Multiplex Real-Time RT-PCR for Simultaneous Detection and Differentiation of Avian Bornaviruses

**DOI:** 10.3390/v13071358

**Published:** 2021-07-13

**Authors:** Brigitte Sigrist, Jessica Geers, Sarah Albini, Dennis Rubbenstroth, Nina Wolfrum

**Affiliations:** 1 Department of Poultry and Rabbit Diseases, Institute for Food Safety and Hygiene, Vetsuisse Faculty, University of Zurich, CH-8057 Zurich, Switzerland; brigitte.sigrist-cathrein@uzh.ch (B.S.); salbini@vetbakt.uzh.ch (S.A.); 2 Institute of Diagnostic Virology, Friedrich-Loeffler-Institut, 17493 Greifswald, Insel Riems, Germany; jessica.geers@web.de (J.G.); dennis.rubbenstroth@fli.de (D.R.); 3 Medical Center, Institute of Virology, University of Freiburg, 79104 Freiburg, Germany

**Keywords:** avian bornavirus, diagnostics, multiplex real-time RT-PCR

## Abstract

Avian bornaviruses were first described in 2008 as the causative agents of proventricular dilatation disease (PDD) in parrots and their relatives (Psittaciformes). To date, 15 genetically highly diverse avian bornaviruses covering at least five viral species have been discovered in different bird orders. Currently, the primary diagnostic tool is the detection of viral RNA by conventional or real-time RT-PCR (rRT-PCR). One of the drawbacks of this is the usage of either specific assays, allowing the detection of one particular virus, or of assays with a broad detection spectrum, which, however, do not allow for the simultaneous specification of the detected virus. To facilitate the simultaneous detection and specification of avian bornaviruses, a multiplex real-time RT-PCR assay was developed. Whole-genome sequences of various bornaviruses were aligned. Primers were designed to recognize conserved regions within the overlapping X/P gene and probes were selected to detect virus species-specific regions within the target region. The optimization of the assay resulted in the sensitive and specific detection of bornaviruses of Psittaciformes, Passeriformes, and aquatic birds. Finally, the new rRT-PCR was successfully employed to detect avian bornaviruses in field samples from various avian species. This assay will serve as powerful tool in epidemiological studies and will improve avian bornavirus detection.

## 1. Introduction

Bornaviruses (family *Bornaviridae*) are enveloped viruses containing a non-segmented single-stranded RNA genome of negative polarity [1,2]. Until 2008, Borna disease virus 1 and 2 (BoDV-1 and -2), causing neurologic diseases in humans, horses, sheep, and other domestic mammals, were the only known members of the family. In 2008, the first avian bornaviruses were discovered in Psittaciformes and shown to be the causative agents of proventricular dilatation disease (PDD) in pet parrots. Sequencing revealed less than 70% nucleotide homology with the mammalian bornaviruses BoDV-1 and -2 [3,4].

PDD occurs worldwide, primarily in captive Psittaciformes. PDD-like diseases have also been reported in birds of additional orders, such as Passeriformes, Anseriformes, Pelicaniformes, Falconiformes, and Piciformes, but association with avian bornaviruses has yet to be confirmed for these birds [5]. The eponymous clinical sign of PDD is a dilatation of the proventriculus with associated gastrointestinal signs thought to be the outcome of virus-induced damage of the enteric nervous system [6]. The gastrointestinal signs are often but not always accompanied by neurological conditions such as tremors, ataxia, seizures, and blindness. Furthermore, neurological disorders can occur in the absence of gastrointestinal signs [7].

**Figure 1:**
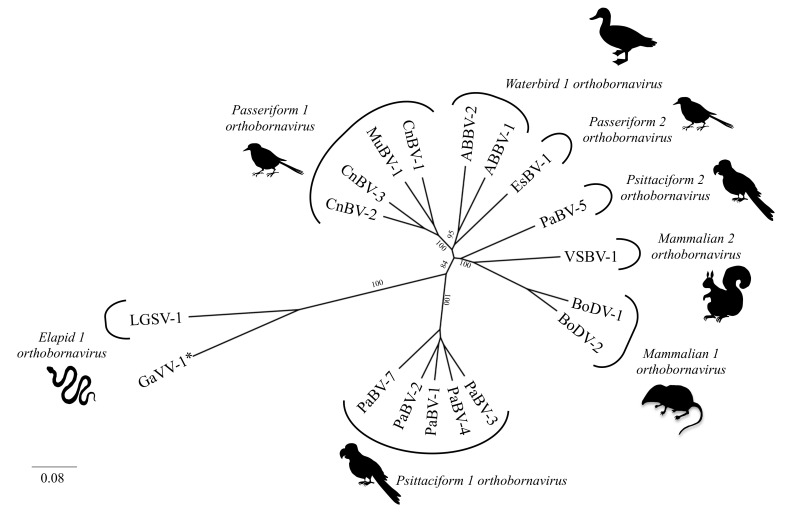
Phylogeny of the genus *Orthobornavirus*. Complete P gene sequences (606 nucleotides) of representative orthobornavirus sequences were analyzed using the neighbor-joining algorithm and the Jukes–Cantor distance model in the Geneious R11 software. Values at branches represent support in 1000 bootstrap replicates. Only boot-strap values ≥70 at major branches are shown. * These viruses have yet not been classified by the International Committee on Taxonomy of Viruses (ICTV). ABBV: aquatic bird bornavirus; BoDV: Borna disease virus; CnBV: canary bornavirus; EsBV: estrildid finch bornavirus; GaVV: Gabon viper virus; LGSV: Loveridge’s garter snake virus; MuBV: munia bornavirus; PaBV: parrot bornavirus; VSBV: variegated squirrel bornavirus.

Following the first detection in 2008, further avian bornaviruses were discovered in psittacines (Psittaciformes), passerines (Passeriformes), and aquatic birds (Anseriformes and Charadriiformes) [8,9]. To date, at least 15 genetically distinct avian bornaviruses are known, forming at least five viral species within the genus *Orthobornavirus* (Figure 1). Eight parrot bornaviruses (PaBV-1 to -8) have been detected in psittacines, belonging to the species *Psittaciform 1 orthobornavirus* (PaBV-1/-2/-3/-4/-7 and -8) and *Psittaciform 2 orthobornavirus* (PaBV-5 and -6). Avian bornaviruses detected in passerine birds belong to the species *Passeriform 1 orthobornavirus* (canary bornavirus 1 to 3, CnBV-1 to -3, and munia bornavirus 1, MuBV-1) and *Passeriform 2 orthobornavirus* (estrildid finch bornavirus 1, EsBV-1). The aquatic bird bornaviruses 1 and 2 (ABBV-1 and -2) constitute the species *Waterbird 1 orthobornavirus* [8,9].

Waterbird 1 orthobornaviruses (ABBV-1, ABBV-2) have been detected in various wild birds, mainly in Anseriformes such as wild geese, including Canada geese (*Branta canadensis*), trumpeter swans (*Cygnus buccinators*), mute swans (*Cygnus olor*), and mallards (*Anas platyrhynchos*) [10,11,12,13,14], but also in Charadriiformes, such as Eurasian oystercatchers (*Haematopus ostralegus*) and gulls [15,16]. Some of the infected birds showed histopathological lesions indicative of bornavirus-induced disease, suggesting that ABBV-1 might be pathogenic for Anseriformes. However, no systematic studies have been published on the correlation between ABBV-1 positive/negative birds and histopathological lesions to establish an association between infection and disease. Likewise, the experimental reproduction of the disease has not been performed. Thus, evidence for the pathogenicity of ABBV-1 in waterfowl is missing [10,13,14,15,17]. Unlike bornaviruses of waterbirds, avian bornaviruses of psittacines and passerines have so far only been detected in captive populations [18,19]. Since the viruses were not found in wild populations, their origin and route of introduction into captive populations remain unclear. Furthermore, it is still not clear how the viruses are transmitted. Vertical and horizontal routes were discussed, but the circumstances of successful transmission remain elusive [15,20,21,22].

Although plenty of research has been conducted in the 12 years since the discovery of avian bornaviruses, details about their epidemiology, routes of transmission, possible introduction into captive populations, and natural reservoirs are still missing. A helpful tool for answering at least some of these questions is precise diagnostic assays for screening various bird populations for avian bornavirus infections. Currently, most rRT-PCR assays are designed to specifically detect only a single avian bornavirus, such as PaBV-2, PaBV-3, PaBV-4, CnBV-2, or ABBV-1 [3,10,23,24,25]. Few rRT-PCRs have been designed for the detection of a small range of closely related viruses, such as the members of the bornaviral species Psittaciform 1 orthobornavirus [26,27]. Schlottau et al. [28] published a set of generic primers and probes for the detection of a broad spectrum of orthobornaviruses. However, data on its potential for the detection of avian bornaviruses have not been provided. Further assays detecting a broad range of bornaviruses are mostly conventional, gel-based RT-PCR assays, which are more laborious and time-consuming. Furthermore, most of these do not recognize all of the as-yet known avian bornaviruses [4,11,18,19,29].

Here, we describe a new multiplex rRT-PCR assay that enables the simultaneous detection and differentiation of various avian bornaviruses. The primers were designed to target conserved regions within the region of the overlapping orthobornavirus X and P genes. They can also be used in a conventional gel-based RT-PCR assay. The triplex rRT-PCR assay includes three different probes, each with a unique reporter dye, designed to detect either parrot bornaviruses of the species *Psittaciform 1 orthobornavirus* (PaBV-1/-2/-3/-4/-7), passerine bornaviruses of the species *Passeriform 1* and *2 orthobornavirus* (CnBV-1/-2/-3, MuBV-1, EsBV-1), or members of the species *Waterbird 1 orthobornavirus* (ABBV-1/-2). The assay was established using in vitro-transcribed orthobornavirus RNAs and validated by a comparative analysis of 69 clinical samples with known avian bornavirus infection status.

## 2. Materials and Methods

### 2.1. Virus Strains and Clinical Samples

One hundred-fold diluted RNA extracted from bornavirus-infected cell cultures or tissue samples of the following avian and mammalian orthobornaviruses was used as reference samples: PaBV-1/-2/-4/-7, CnBV-1/-2/-3, EsBV-1, ABBV-1, and BoDV-1/-2 (Table S1). The RNA extracted from 82 clinical samples originating from various psittacine, passerine, and aquatic bird species were used for the validation of the assay (Table S2). The avian bornavirus status of the samples was known based on previous conventional or real-time RT-PCR testing and the subsequent identification of the virus by sequence analysis, as described previously [15,18,19,30,31].

### 2.2. Primer and Probe Design

**Figure 2:**
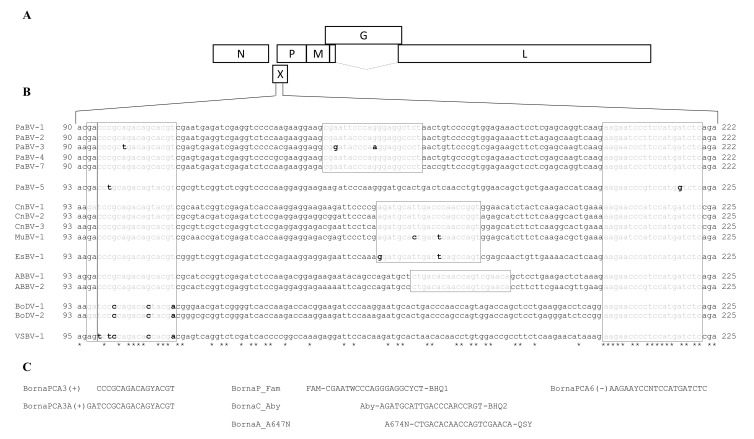
Primer and probe design. (**A**). Schematic presentation of the orthobornavirus genome. (**B**). Alignment of the partial orthobornavirus X/P gene sequences (positions 90/93 to 222/225). Boxes: sequences corresponding to the primer and probe binding regions; grey: nucleotides matching the primer/probe sequence; black, boldface: nucleotides mismatching with the primer/probe sequence. PaBV-6 and PaBV-8 are not included in this alignment, since no X gene sequences are available for these viruses. (**C**). Primer and probe sequences.

For primer and probe design, whole-genome sequences of at least one representative of all known avian and mammalian orthobornaviruses were aligned using Clustal Omega (Table S1) [32]. The overlapping region of the viral X and P genes contained highly conserved sequences, enabling the manual design of two variants of a degenerate forward primer and a single degenerate reverse primer, allowing the amplification of a wide range of avian orthobornaviruses (Figure 2). Furthermore, the selected region also contained stretches with variable regions, enabling the differentiation of avian bornavirus groups by specific probes. Three probes were designed for the detection of either psittacine bornaviruses of the species *Psittaciform 1 orthobornavirus* (PaBV-1/-2/-4/and -7; probe BornaP_Fam), passerine bornaviruses (CnBV-1/-2/-3, and EsBV-1; probe BornaC_Aby), or aquatic bird bornaviruses (ABBV-1 and -2; probe BornaA_A647N). Primers and probes were synthesized by Microsynth (Balgach, Switzerland) or Applied Biosystems (Thermo Fisher Scientific, Waltham, MA, USA). To allow for the simultaneous usage of all three probes, different reporter fluorophores were chosen for each of the probes (Figure 2). The basic local alignment search tool (BLAST) from the National Center for Biotechnology Information (NCBI) was applied with default settings to confirm the specificity of the probes, and this revealed no off targets. Merely, probe BornaC_Aby, targeting the bornaviruses of Passeriformes, showed a 90% nucleotide identity with the respective sequence of BoDV-1.

### 2.3. Cloning of Recombinant Plasmids and Preparation of RNA Reference Transcripts

The viral RNA of reference samples was reverse transcribed into cDNA using the Reverse Transcription System (Promega, Madison, WI, USA) according to the manufacturer’s protocol using a mix of random hexamer and Oligo(dT)15 primers. The complete X gene of 11 orthobornaviruses (PaBV-1/-2/-4/-7, CnBV-1/-2/-3, EsBV-1, ABBV-1, BoDV-1/-2) was amplified (primers used are presented in Table S3) and cloned into the pCR2.1-TOPO vector (Invitrogen, Thermo Fisher Scientific, Waltham, MA, USA). Successful cloning was verified by sequencing (Microsynth, Balgach, Switzerland). The vector constructs were linearized by BamHI (NEB, Ipswich, MA, USA) digestion and in vitro-transcribed from the T7 promoter using the MEGAscript T7 kit (Ambion, Thermo Fisher Scientific, Waltham, MA, USA), according to the manufacturer’s instructions, but with an overnight incubation period at 37 °C. The in vitro-transcribed RNA was treated with Nb.BssSI (NEB, Ipswich, MA, USA) and thereafter with Turbo DNase (Ambion, Thermo Fisher Scientific, Waltham, MA, USA) and further processed by a LiCl precipitation. The RNA concentration was determined spectrophotometrically using the NanoDropTM 2000 Spectrophotometer (Thermo Fisher Scientific, Waltham, MA, USA) for the calculation of copy numbers. Copy numbers were calculated with the following formula: Copy No. = (mass RNA [g]/mol. weight [g/mol]) x Avogadro’s Number. The absence of DNA from the in vitro-transcribed RNA was assessed using a real-time PCR (excluding the RT step) with 10-fold serial dilutions of the RNA (data not shown).

### 2.4. Conventional RT-PCR Assays

The newly designed degenerate primers targeting the X gene were first employed in a conventional one-step RT-PCR (Xcon os), amplifying a product of 125 base pairs (bp). Defined copy numbers of in vitro-transcribed recombinant plasmids encoding the X gene of various bornaviruses were used as templates to determine the detection limit of the assay for the different viruses. Each reaction contained 1x reaction mix, 0.25 µL RT-mix (both QuantiTect Probe RT-PCR Master Mix, Qiagen, Hilden, Germany), 0.25 µM of each primer, 1 µL RNA, and diethyl pyrocarbonate (DEPC)-treated H_2_O in a total volume of 25 µL. The PCR was performed using the AllInOne-cycler (Bioneer, Daejeon, South Korea) with the following cycler setup: 50 °C for 30 min, 95 °C for 15 min, 45 cycles of 94 °C for 1 min, 53 °C for 1 min, 72 °C for 1 min, and a final extension at 72 °C for 10 min. Amplification products were subjected to capillary electrophoresis using the QIAxcel Advanced system (Qiagen, Hilden, Germany).

In addition, the new primer set targeting the X gene (Xcon) was compared to three different degenerate primer sets targeting the consensus sequences of the M gene (Mcon, Mcon-W) or N gene (Ncon) of various avian- and mammalian bornaviruses (Table S4) [4,29]. These primers were designed to amplify a 360 bp or 352 bp fragment of the bornavirus M gene and a 398 bp fragment of the bornavirus N gene. Since in vitro-transcribed RNA preparations with known copy numbers were not available for the N and M genes, the cDNA of serial half-logarithmic dilutions of reference RNAs originating from bornavirus-infected cell cultures or tissue samples were used as templates for the comparative analysis. The initial (=highest) concentration of cDNA used was 200 ng. Each reaction contained 1x Taq buffer, 0.625 U Taq, 3 mM MgCl2 (Qiagen, Hilden, Germany), 0.2 µM of primer set Ncon and Mcon, or 0.4 µM of primer set Mcon-W, or 0.25 µM primer set X, 5 µL cDNA, and diethyl pyrocarbonate (DEPC)-treated H2O in a total volume of 25 µL. The amplification conditions were as follows: 1× 94 °C for 5 min, 45 cycles of 94 °C for 1 min, 48.5 °C for 30 s (Mcon, Ncon) or 55 °C for 30 s (Mcon-W) or 53 °C for 30 s Xcon), 72 °C for 1 min, and a final extension at 72 °C for 5 min.

### 2.5. Real Time RT-PCR Assays

One-step rRT-PCR reactions were performed for the newly designed X gene primers (multiBornaX) with either an individual probe or all three probes combined (Figure 2). The 25 µL reaction contained 1× reaction mix, 0.25 µL RT-mix (both QuantiTect Probe RT-PCR Master Mix, Qiagen, Hilden, Germany), 0.25 µM of each primer and probe, 1 µL in vitro-transcribed reference RNA or 2.5 µL RNA extracted from clinical samples in DEPC-treated H_2_O. Reverse transcription and amplification were performed on a QuantStudio 5 Real-Time PCR System (Applied Biosystems, Thermo Fisher Scientific, Waltham, MA, USA) with the following conditions: 1× 50 °C for 30 min, 1× 95 °C for 10 min, 45 cycles of 95 °C for 15 s, 53 °C for 1 min, 70 °C for 1 min.

Clinical samples were tested with the newly designed multiplex rRT-PCR assay in comparison with previously published and newly designed bornavirus rRT-PCRs. All samples were tested by the assay panBorna_7.2, designed for the detection of a broad range of orthobornaviruses [28]. The assays PaBVcon_MD [10] and PaBVcon_PG [26], both designed for the detection of the members of *Psittaciform 1 orthobornavirus*, PaBV-4_P [3] and PaBV-2_P [24], were employed for psittacine samples. Samples from Passeriformes (canaries and estrildid finches) were tested by CnBV-2_P [23]. Two new primer/probe combinations were designed for the detection of aquatic bird bornaviruses. ABBV-1_M was based on the assay published by Delnatte et al. [10] and modified to also match European ABBV-1 sequences. ABBVcon_P was designed to target the consensus sequence of all available ABBV-1 and ABBV-2 sequences. Primer and probe sequences are provided in Table S4. The RT-qPCRs were performed using the qScript XLT one-step RT-qPCR ToughMix Kit (Quanta BioSciences, Gaithersburg, MD, USA). Each reaction of 12.5 µL contained 6.25 μL of 2× qScript XLT One-Step RT-qPCR ToughMix, primers and probes at the final concentrations provided in Table S4, and 2.5 μL of RNA template. The RT-qPCRs were performed on a CFX96 Real-Time PCR Detection System (Bio-Rad, Hercules, CA, USA) with the following cycler setup: 50 °C for 10 min and 95 °C for 1 min, followed by 45 cycles of 95 °C for 10 s, 57 °C for 30 s, and 68 °C for 30 s.

### 2.6. Determination of Analytical Sensitivity and Specificity

The analytical sensitivity and specificity of the multiBornaX assay were determined by performing the rRT-PCR using either individual X gene probes or as a multiplex assay (all three probes together) with the defined copy numbers of the in vitro-transcribed reference RNAs (10-fold serial dilutions corresponding to 10^0^ to 10^6^ copies per reaction). Two independent experiments were performed in duplicate. Standard curves were generated with the mean C_T_ values of both experiments and regression analyses were performed using Excel.

To evaluate the potential impact of the presence of multiple RNAs of different avian bornaviruses on the assay performance, the multiBornaX assay was performed with 10^6^ copies of various combinations (3 RNAs per reaction) of in vitro-transcribed reference RNAs.

### 2.7. Statistical Analysis

Positive percent agreement (PPA), negative percent agreement (NPA), and overall percent agreement (OPA) were calculated along with the Wilson score confidence intervals, mean values, percentages, and standard deviations. Student’s *t*-tests were calculated using Excel. *p* < 0.05 was considered to indicate significant differences. PPA, NPA, and OPA were calculated in comparison to all non-standard tests together—i.e., samples for which at least one rRT-PCR was positive were positive, samples for which all rRT-PCRs were negative were negative.

## 3. Results

### 3.1. Conventional RT-PCR and Comparison with Published Primers Detecting a Broad Range of Bornaviruses

For the new multiplex rRT-PCR, X gene primers were designed to detect a broad range of avian bornaviruses. These primers can also be used in a conventional gel-based RT-PCR, which might be beneficial for diagnostic facilities without access to a real-time cycler. Using defined copy numbers of in vitro-transcribed reference RNAs encoding the X genes of various bornaviruses resulted in the detection of all nine avian bornavirus RNAs with down to 10^2^ copies per reaction for PaBV-1/-4/-7 and CnBV-2; 10^3^ copies for PaBV-2, CnBV-1/-3, and EsBV-1; and 10^4^ copies for ABBV-1. In contrast, BoDV-1 required 10^8^ copies for a positive result and BoDV-2 was not detected at all (Table 1).

The new degenerate primers targeting the X gene (Xcon) were compared to previously published degenerate primer sets targeting the consensus sequences of the M gene (Mcon, Mcon-W) or N gene (Ncon) of various avian and mammalian bornaviruses [4,29]. Since in vitro-transcribed RNA with defined copy numbers of N and M genes were not available, a dilution series of cDNA reverse-transcribed from RNAs from infected cell cultures or tissue samples (Supplemental Table S1) was used for comparative analysis. The Ncon and the new Xcon RT-PCR revealed the highest analytical sensitivity for a broad range of avian bornaviruses (Table 1). While the Xcon assay was the only assay detecting the ABBV-1-positive RNA preparation, it did not recognize any of the available mammalian bornavirus samples. Under the applied conditions, the Mcon-W RT-PCR established by Weissenböck et al. [29] was the least sensitive. It only detected seven out of nine avian bornaviruses and none of the mammalian bornaviruses (Table 1).

### 3.2. Analytical Sensitivity and Specificity of the Avian Bornavirus Multiplex rRT-PCR Assay

**Figure 3:**
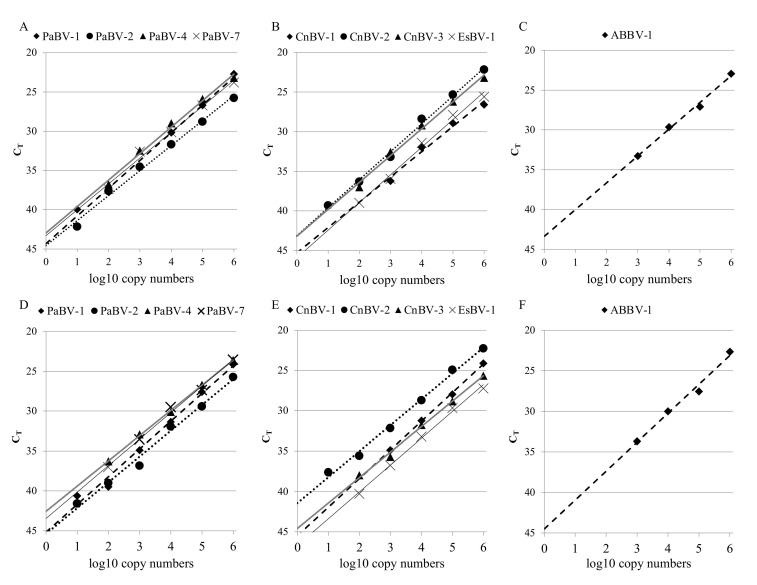
Analytical sensitivity of the rRT-PCR assay. Serial ten-fold dilutions of quantitated in vitro-transcribed reference RNAs of different avian bornaviruses were used as templates in a singleplex assay with the respective probe ((**A**) BornaP_Fam; (**B**) BornaC_Aby; (**C**) BornaA_A647N) or in a multiplex assay where all three probes were used simultaneously (**D**–**F**). Results are presented as the mean C_T_ values of two independent experiments.

The analytical sensitivity of the multiBornaX assay was first evaluated by performing rRT-PCR using each of the three X gene probes separately with serial tenfold dilutions of quantitated in vitro-transcribed reference RNAs (Figure 3A–C; Table 2). Using probe BornaP_FAM, between 10^1^ and 10^2^ copies of PaBV-1/-2/-4/-7 RNA were detected. Probe BornaC_Aby recognized between 10^1^ and 10^3^ copies of CnBV-1/-2/-3 and EsBV-1, and probe BornaA_A647N detected 10^3^ copies of ABBV-1 per reaction. BoDV-1 and BoDV-2 were not detected when using any of the three probes (data not shown). The efficiency (E), which is the rate of amplicon generation, was between 91.8% and 107.3%, and the coefficient of determination (r^2^), reflecting the linearity of the standard curve, ranged from 0.980 to 0.995 (Table 2).

Subsequently, the multiBornaX assay was performed using the three X gene probes simultaneously with the same reference RNA dilution series (Figure 3D–F; Table 2). The analytical sensitivity of the multiplex assay for the desired viruses was unaffected when compared to the respective singleplex assay. The efficiency (E) was between 90.9% and 107.6% and the coefficient of determination (r^2^) ranged from 0.983 to 0.999 (Table 2). Furthermore, the test confirmed the specificity of each of the three probes, since the psittacine bornavirus RNAs (PaBV-1/-2/-4/-7) were exclusively detected with probe BornaP_Fam, probe BornaA_A647N specifically detected the ABBV-1 RNA, and probe BornaC_Aby solely detected the four tested passerine bornavirus RNAs (CnBV-1/-2/-3, EsBV-1; data not shown).

To assess whether the simultaneous presence of multiple avian bornaviruses interferes with the detection of the respective viruses, combinations of 10^6^ copies of various in vitro-transcribed bornavirus reference RNAs were used as templates in the multiplex rRT-PCR. As shown in Table S5, all bornavirus RNAs were detected independent of the presence of other bornavirus RNAs. However, the presence of multiple avian bornavirus RNAs resulted in significantly higher C_T_ values for the detection of PaBV-2/-4 and CnBV-1, whereas significantly lower C_T_ values were achieved for PaBV-7 and EsBV-1. There was no impact on the detection of PaBV-1, CnBV-2/-3, and ABBV-1.

### 3.3. Detection of Avian Bornavirus RNA in Field Samples

The herein-described multiBornaX assay was used to detect avian bornavirus RNA in 82 clinical samples with known avian bornavirus status. Since there is no gold standard for the detection of avian bornaviruses, the new assay was compared to the previously published assay panBorna_7.2 [28] as well as a variety of assays for a more specific detection of particular avian bornavirus subsets (Table S2).

Applying the multiBornaX assay, 77 samples were correctly identified with the respective bornavirus probe. Three of these samples (#58, #70, #73) were considered weakly positive, since the C_T_ values were above 39. No positive off-target results were observed with the three probes (Table 3), allowing for the simultaneous assessment of the present virus type. The panBorna_7.2 assay provided correct results for 75 out of 82 tested samples. In both assays, false-negative results were observed for those samples that tested weakly positive in the respective more targeted assays with C_T_ values around 30 or higher (#71, #72, #77), while strongly positive samples (C_T_ < 21) were consistently detected (Table S2).

Since the sample size for some bornaviruses was rather small (*n*_AABV-1_ = 5, *n*_PaBV-2_ = 5), the new multiBornaX assay was not compared to the individual tests but to all eight non-standard avian bornavirus rRT-PCR assays together. This revealed a positive percentage agreement of 92.8% (95% CI 84.1–96.9), a negative percentage agreement of 100% (95% CI 77.2–100), and an overall percentage agreement of 93.9% (95% CI 86.5–97.4).

PaBVcon_MD [10] and PaBVcon_PG [26] readily detected PaBV-1/-2/-4 and -7 (Table 4, Table S2). PaBV-2_P [24], which was modified from PaBVcon_PG to allow for the better detection of PaBV-2, did not prove beneficial as compared to the original assay (Table S2). PaBV-4_P [3] detected PaBV-4 and to some extent PaBV-1, but not PaBV-2 and PaBV-7 (Table 4, Table S2). CnBV-2_P [23] also detected passerine bornaviruses other than CnBV-2 (Table 4, Table S2). The two assays designed for the detection of aquatic bird bornaviruses, ABBV-1_M and ABBVcon_P, correctly detected all five available ABBV-1-positive samples (Table 4).

## 4. Discussion

The aim of this project was to establish an rRT-PCR enabling the simultaneous identification and differentiation of avian bornaviruses. The first goal was the design of primers that could detect a broad range of (avian) orthobornaviruses. A conventional RT-PCR using these new primers was shown to detect the RNAs of ten available orthobornaviruses (nine avian bornaviruses and the mammalian virus BoDV-1) with varying sensitivity. Since RNA preparations of ABBV-2, PaBV-3, -5, -6, -8, MuBV-1, and VSBV-1 were not available, their detection could not be validated experimentally.

When comparing the new Xcon assay with previously published conventional RT-PCR assays [4,29], all of them revealed a broad reactivity for most of the tested avian bornaviruses, albeit with variable sensitivity. This is in agreement with previous work [18,19]. The Xcon assay provided the highest sensitivity for the majority of the tested avian bornavirus cDNAs, particularly for CnBV-1/-2/-3 and ABBV-1. In contrast, Ncon and Mcon were more sensitive for the two tested mammalian bornaviruses.

Using the herein described X-specific primers as part of the multiBornaX assay with TaqMan probes specifically targeting groups of either psittacine, passerine, or waterbird bornaviruses, the detection limit was even reduced by one or two log units for six out of nine tested avian bornaviruses, whereas it was unchanged for the remaining three viruses. The sensitivity did not differ between singleplex assays using each probe individually and the multiplex assay simultaneously including all three probes. The efficiency (singleplex: 91.8 to 107.3%; multiplex: 90.9 to 107.6%) and the linearity (r^2^ value; singleplex: 0.980 to 0.995; multiplex: 0.983 to 0.999) were both in the desired range for rRT-PCR assays [33,34].

It was striking that the consistently lowest analytical sensitivity was for ABBV-1. This effect was independent of the sample used (RNA or cDNA) and independent of the test (conventional PCR or rRT-PCR; singleplex, multiplex). A possible explanation could be the different secondary structures of the amplified region. The analysis (UNAfold, www.idtdna.com, accessed on 1 July 2021, default settings) of the respective regions showed significantly lower ΔG values for PaBV-1/-2/-4/-7, CnBV-2/-3, and EsBV-1 (between −12.51 and −9.84 kcal/mole) compared to ABBV-1 and CnBV-1 (−7.82 and −5.98, respectively). At least in the rRT-PCR assay, CnBV-1 had the same analytical sensitivity as ABBV-1, which speaks for the influence of the secondary structure on the sensitivity of the PCR.

The herein-developed primer/probe-set was able to detect all nine available avian bornaviruses in a conventional as well as in an rRT-PCR assay. However, in silico sequence analysis suggests that the primers also allow for the detection of additional bornaviruses using the Xcon assay. The primer target sequences of MuBV-1 and ABBV-2 share 100% identity with their next relatives (ABBV-1, and CnBV-1/-2/-3, respectively), which were readily detected using the newly designed primers. ABBV-2 also does not show mismatches for the probe Borna_A, suggesting the successful detection by the multiBornaX assay too. In contrast, the sequence of MuBV-1 contains two mismatches with probe Borna_C. However, since EsBV-1 is detected using this probe despite two mismatches, MuBV-1 is likely to be detected by the rRT-PCR. The PaBV-3 sequence contains one mismatch with each of the two forward primer variants and two mismatches with the probe Borna_P, which may still allow for detection. The X gene of PaBV-5, belonging to the species *Psittaciform 2 orthobornavirus* [15], shares only about 75% sequence identity with the other avian bornaviruses. While the primer sequences show one mismatch in each of the two forward primer variants and in the reverse primer, none of the three probes show any similarity to the respective PaBV-5 sequences. Thus, PaBV-5 is likely to be detected by conventional RT-PCR but not by rRT-PCR assay. The sequences of BoDV-1 and BoDV-2 possess three mismatches with the forward primer. While BoDV-1 was amplified in the conventional assay with an extremely low sensitivity, BoDV-2 was not. Neither BoDV-1 nor BoDV-2 were detected in the rRT-PCR assays. The detection of VSBV-1, belonging to the species *Mammalian 2 orthobornavirus,* is unlikely due to five mismatches in the forward primer. No X gene sequences were available for PaBV-6 and PaBV-8.

To analyze whether the presence of multiple RNAs of different avian bornaviruses has an impact on their detection, combinations of three different avian bornavirus reference RNAs were used as targets in the multiBornaX assay. All the avian bornaviruses were detected in all tested combinations. C_T_ values of some samples (higher C_T_ values for PaBV-2/-4, CnBV-1, lower C_T_ values for PaBV-7, EsBV-1) differed significantly when compared with C_T_ values obtained in the absence of other bornavirus RNA. It can only be speculated as to why changes in C_T_ values occur in the presence of multiple targets. The primers used allowed for the detection of all the herein applied RNA targets. However, both forward and reverse primers contain wobble nucleotides, which may lead to the favorability of one or the other target and thus result in a competition for the primers. Nevertheless, these differences are most likely negligible. Even though, it has been shown that occasional co-infections with different viruses of the same species occur, such as PaBV-4 with PaBV-2 or PaBV-7 [29,30], no co-infections of a particular individual with different avian bornavirus species have been detected so far. On the other hand, it cannot be ruled out that co-infections with various bornavirus species may occur. Thus far, no systematic studies have been conducted to address this question. This would also be interesting in order to gain possible insights into bornavirus distribution and transmission. In this context, the new multiplex rRT-PCR will be a valuable tool, since it enables the time-efficient screening of samples for the presence of various bornavirus species.

Using the new multiBornaX assay, 77 out of 82 field samples with known avian bornavirus status were correctly identified as positive or negative (93.9%), and this is comparable to the panBorna_7.2 assay, which correctly identified 75 out of 82 analyzed samples (91.5%). However, the new multiplex rRT-PCR has the advantage of the simultaneous differentiation of different types of bornaviruses.

The new multiBornaX test was compared to all comparative tests together and resulted in a high percentage of positive agreement (92.8%) and 100% negative percentage agreement. Each of the comparative assays has its strengths and weaknesses, which makes direct comparison difficult and could lead to biased results. In addition, some viruses were represented in very low numbers (*n*_AABV-1_ = 5, *n*_PaBV-2_ = 5), which would also have reduced the significance of a direct comparison of the tests.

The respective avian bornaviruses were not uniformly represented within the studied sample collection. More than two thirds of the bornavirus-positive samples represented PaBV-4 (30.4%), CnBV-1 (20.3%), and CnBV-2 (20.3%), which is in line with the relatively wide distribution of these viruses in psittacine or canary holdings [15]. All samples of PaBV-1/-2/-7, CnBV-1/-2/-3, and EsBV-1 were identified correctly with the new multiplex rRT-PCR assay. Three out of the five false negative samples were PaBV-4, and one was ABBV-1. The fifth false negative sample originated from a seropositive macaw. The swab tested weakly positive only in the PaBV-4_P assay, but sequence analysis for the definitive identification of the virus failed.

Assays designed to specifically target a single bornavirus or a small group of closely related bornaviruses were still superior in detecting the respective viruses compared to both broad range assays, particularly for the detection of PaBV-4 and ABBV-1.

The origin of the samples (swabs, organs) had no impact on the rRT-PCR results. Nevertheless, it cannot be excluded that swab samples from animals secreting low amounts of virus will not be detected by the multiBornaX assay.

The herein presented multiBornaX assay was shown to allow for the simultaneous detection and differentiation of avian bornaviruses of at least four viral species. It was shown to be sensitive and specific. Therefore, it will be a valuable tool for diagnostic routine as well as for epidemiologic avian bornavirus research.

## Figures and Tables

**Table 1:** Conventional RT-PCR assay for the detection of orthobornaviruses. Detection limit and comparative analysis.

Virus	Detection Limit of Xcon	Comparative Analysis of RT-PCR Assays (Highest log_10_ Dilution Factor of Reference cDNA with pos. Result) ^b^			
	(Copy Numbers per Reaction) ^a^	Xcon	Ncon [4]	Mcon [4]	Mcon-W [24]
PaBV-1	10^2^	3.0	2.0	3.0	2.0
PaBV-2	10^3^	4.0	4.0	3.5	2.0
PaBV-4	10^2^	3.0	3.0	2.5	2.0
PaBV-7	10^2^	3.5	3.0	2.5	2.0
CnBV-1	10^3^	3.0	2.5	0.5	0.5
CnBV-2	10^2^	2.0	0.5	2.5	1.5
CnBV-3	10^3^	2.5	0.5	1.0	0.5
EsBV-1	10^3^	0.5	1.0	0.5	nd
ABBV-1	10^4^	0.5	nd	nd	nd
BoDV-1	10^8^	nd	2.0	0.5	nd
BoDV-2	nd	nd	1.5	0.5	nd

nd: not detectable. ^a^ Serial ten-fold dilutions of in vitro-transcribed X gene RNA with known copy numbers were analyzed. ^b^ Serial half-logarithmic dilutions of cDNA reverse transcribed from RNA samples of reference viruses were analyzed. Results are presented as means of two independent experiments.

**Table 2:** Real-time RT-PCR assays: Sensitivity and regression analyses of standard curves.

Parameter	Probe Target	BornaP_FAM				BornaC_Aby				BornaA_A674N
		PaBV-1	PaBV-2	PaBV-4	PaBV-7	CnBV-1	CnBV-2	CnBV-3	EsBV-1	ABBV-1
r^2^	singleplex	0.993	0.993	0.992	0.990	0.980	0.995	0.992	0.990	0.993
	multiplex	0.987	0.989	0.999	0.993	0.999	0.994	0.995	0.996	0.983
E	singleplex	91.8%	106.1%	98.0%	100.9%	107.3%	92.1%	97.3%	93.5%	95.2%
	multiplex	94.1%	104.1%	107.4%	100.3%	91.3%	104.8%	107.6%	99.9%	90.9%
Detection limit ^a^	singleplex	10^1^	10^1^	10^2^	10^2^	10^3^	10^1^	10^2^	10^2^	10^3^
	multiplex	10^1^	10^1^	10^2^	10^2^	10^3^	10^1^	10^2^	10^2^	10^3^

r^2^, coefficient of determination; E, efficiency. ^a^ lowest copy number that was reproducibly detected.

**Table 3:** Impact of the presence of multiple avian bornavirus RNAs on the rRT-PCR performance. Comparison between multiple RNA targets vs. a single target RNA (10^6^ copies per reaction).

	Probe Target	PaBV-1	PaBV-2	PaBV-4	PaBV-7	CnBV-1	CnBV-2	CnBV-3	EsBV-1	ABBV-1
**mean** **C_T_**	**st**	24.15 ±0.19	25.72 ±0.36	23.61 ±0.55	23.55 ±0.49	24.11 ±0.97	22.24 ±0.89	25.64 ±0.80	27.20 ±0.67	22.64 ±0.33
	**mt**	24.04 ±0.15	29.36 ±0.34	25.26 ±0.18	21.99 ±0.20	28.19 ±0.68	23.52 ±0.84	25.49 ±0.91	24.45 ±0.87	23.72 ±1.03
***p*-value**		0.5912	0.0002	0.0155	0.0170	0.0409	0.2299	0.8026	0.0130	0.1035

Results are presented as average of four C_T_ values +/− standard deviation. mt, multiple targets; st, single target. *p* < 0.05 was considered to indicated significant differences (analysed by Student’s *t*-test). st, single target; mt, multiple targets.

**Table 4:** Comparative testing of 82 clinical samples from various avian hosts with different rRT-PCR assays for the detection of avian orthobornaviruses. Total numbers of tested and positive samples are provided.

Sample Origin Virus	Sample Number	multiBornaX			panBorna 7.2	PaBVcon MD	PaBVcon PG	PaBV-2 P	PaBV-4 P	CnBV-2 P	ABBV-1 M	ABBVcon P
		Probe P	Probe C	Probe A								
Psittaciformes												
PaBV-1	2	2	0	0	2	2	2	2	2	-	-	-
PaBV-2	5	5	0	0	5	5	5	5	0	-	-	-
PaBV-4	21	18 ^a^	0	0	16	20	19	19	21	-	-	-
PaBV-4 & -7 ^b^	2	2	0	0	2	2	2	2	2	-	-	-
PaBV-7	1	1	0	0	1	1	1	1	0	-	-	-
undetermined	1	0	0	0	0	0	0	0	1	-	-	-
negative	5	0	0	0	0	0	0	0	0	-	-	-
Passeriformes												
CnBV-1	14	0	14	0	14	-	-	-	-	11	-	-
CnBV-2	14	0	14	0	14	-	-	-	-	14	-	-
CnBV-3	2	0	2	0	2	-	-	-	-	2	-	-
EsBV-1	2	0	2	0	2	-	-	-	-	2	-	-
negative	4	0	0	0	0	-	-	-	-	0	-	-
aquatic birds												
ABBV-1	5	0	0	4	3	-	-	-	-	-	5	5
negative	4	0	0	0	0	-	-	-	-	-	0	0

-: not determined, ^a^ Three out of 18 positive results revealed C_T_ values >39, ^b^ Samples originating from a cockatoo with PaBV-4 and PaBV-7 coinfection [25].

## Data Availability

Not applicable.

## Supplementary Materials

The following are available online at https://www.mdpi.com/article/10.3390/v13071358/s1: Table S1: Reference sequences and reference samples used for assay design and generation of in vitro-transcribed RNA standards. Table S2: Comparative rRT-PCR analysis of known avian orthobornavirus positive clinical samples. Table S3: Primers used for the amplification of the X gene reference sequences. Table S4. Primers and probes for conventional and rRT-PCR assays for the detection of orthobornaviruses. Table S5. Impact of the presence of multiple avian bornavirus RNAs on the rRT-PCR performance.
