# Supplementary material for: A New Multiplex Real-Time RT-PCR for Simultaneous Detection and Differentiation of Avian Bornaviruses
Source: Viruses. 2021 Jul 13;13(7):1358. doi: 10.3390/v13071358 (PMC8310230; doi:10.3390/v13071358)
Supplement: Supplementary file 1 [file viruses-13-01358-s001.zip › viruses-1257193-si final.pdf]

Table S1. Reference sequences and reference samples used for assay design and generation of *in vitro*-transcribed RNA standards.
PaBV: parrot bornavirus, CnBV: canary bornavirus, EsBV: estrildid finch bornavirus, MuBV: munia bornavirus, ABBV: aquatic bird bornavirus, BoDV: Borna disease virus, VSBV: variegated squirrel bornavirus

Species	Virus	Sequence used for alignment (Accession no.)	Reference material	
			Isolate/ Strain	Accession no.
Psittaciform 1 orthobornavirus	PaBV-1	NC_030687	#16234	JX065207
	PaBV-2	NC_028106	#17684	JX065197
	PaBV-3	FJ169440	-	-
	PaBV-4	JX065209	#6758	JX065209
	PaBV-7	NC_030689	#16667a	JX065210
Psittaciform 2 orthobornavirus	PaBV-5	KT378600	-	-
Passeriform 1 orthobornavirus	CnBV-1	NC_030690	#7293	KC464471
	CnBV-2	NC_027892	#15864	KC464478
	CnBV-3	NC_024296	VS-4424	KC595273
	MuBV-1	DC292974	-	-
Passeriform 2 orthobornavirus	EsBV-1	NC_038268	VS-4707	NC_038268
Waterbird 1 orthobornavirus	ABBV-1	NC_029642	AF-168	KU748788
	ABBV-2	NC_030691	-	-
Mammalian 1 orthobornavirus	BoDV-1	AJ311522	He/80/FR	AJ311522
	BoDV-2	AJ311524	No/98	AJ311524
Mammalian 2 orthobornavirus	VSBV-1	MF597762	-	-

Table S2. Comparative rRT-PCR analysis of known avian orthobornavirus positive clinical samples.

-: not determined, neg: negative, values in brackets: (C_T value > 39), ns: not specified, pv: proventriculus, gi: gizzard, p/c: pharynx/cloaca, cc: cell culture

Part 1

No.	Common name	Scientific name	Sample	Virus[a]	multiBornaX			panBorna	PaBVcon	PaBVcon	PaBV-2	PaBV-4	CnBV-2	ABBV-1	ABBVcon
					probe P	probe C	probe A	7.2	MD	PG	P	P	P	M	P
**1**	Oystercatcher	*Haematopus ostralegus*	intestine	ABBV-1	neg	neg	38.0	neg	-	-	-	-	-	22.9	22.8
**2**	Oystercatcher	*Haematopus ostralegus*	liver	ABBV-1	neg	neg	neg	29.3	-	-	-	-	-	26.2	24.6
**3**	Mute swan	*Cygnus olor*	swab	ABBV-1	neg	neg	38.6	neg	-	-	-	-	-	31.0	31.1
**4**	Mute swan	*Cygnus olor*	brain	ABBV-1	neg	neg	17.7	16.2	-	-	-	-	-	12.5	12.1
**5**	Mute swan	*Cygnus olor*	brain	ABBV-1	neg	neg	27.1	24.8	-	-	-	-	-	23.5	22.2
**6**	Mute swan	*Cygnus olor*	p/c swab	neg	neg	neg	neg	neg	-	-	-	-	-	neg	neg
**7**	Mallard	*Anas platyrhynchos*	p/c swab	neg	neg	neg	neg	neg	-	-	-	-	-	neg	neg
**8**	Mallard	*Anas platyrhynchos*	p/c swab	neg	neg	neg	neg	neg	-	-	-	-	-	neg	neg
**9**	Domestic goose	*Anser anser domesticus*	organ pool	neg	neg	neg	neg	neg	-	-	-	-	-	neg	neg
**10**	Domestic canary	*Serinus canaria domestica*	brain	CnBV-1	neg	19.6	neg	14.1	-	-	-	-	20.4	-	-
**11**	Domestic canary	*Serinus canaria domestica*	brain	CnBV-1	neg	16.0	neg	13.1	-	-	-	-	24.5	-	-
**12**	Domestic canary	*Serinus canaria domestica*	brain	CnBV-1	neg	18.3	neg	15.1	-	-	-	-	27.1	-	-
**13**	Domestic canary	*Serinus canaria domestica*	organ pool	CnBV-1	neg	24.7	neg	15.0	-	-	-	-	20.5	-	-
**14**	Domestic canary	*Serinus canaria domestica*	organ pool	CnBV-1	neg	35.9	neg	28.5	-	-	-	-	neg	-	-
**15**	Domestic canary	*Serinus canaria domestica*	swab	CnBV-1	neg	35.8	neg	29.3	-	-	-	-	neg	-	-
**16**	Domestic canary	*Serinus canaria domestica*	swab	CnBV-1	neg	30.5	neg	25.9	-	-	-	-	neg	-	-
**17**	Domestic canary	*Serinus canaria domestica*	brain	CnBV-1	neg	20.6	neg	12.9	-	-	-	-	28.3	-	-
**18**	Domestic canary	*Serinus canaria domestica*	brain	CnBV-1	neg	17.2	neg	12.9	-	-	-	-	26.1	-	-
**19**	Domestic canary	*Serinus canaria domestica*	brain	CnBV-1	neg	19.6	neg	16.6	-	-	-	-	27.6	-	-
**20**	Domestic canary	*Serinus canaria domestica*	brain	CnBV-1	neg	21.9	neg	14.6	-	-	-	-	24.9	-	-
**21**	Domestic canary	*Serinus canaria domestica*	brain	CnBV-1	neg	20.9	neg	14.6	-	-	-	-	26.5	-	-
**22**	Domestic canary	*Serinus canaria domestica*	brain	CnBV-1	neg	23.9	neg	14.5	-	-	-	-	25.2	-	-
**23**	Domestic canary	*Serinus canaria domestica*	brain	CnBV-1	neg	21.0	neg	15.3	-	-	-	-	24.3	-	-
**24**	Domestic canary	*Serinus canaria domestica*	organ pool	CnBV-2	neg	37.5	neg	14.8	-	-	-	-	10.8	-	-
**25**	Domestic canary	*Serinus canaria domestica*	swab	CnBV-2	neg	32.4	neg	25.4	-	-	-	-	22.4	-	-
**26**	Domestic canary	*Serinus canaria domestica*	swab	CnBV-2	neg	33.5	neg	26.5	-	-	-	-	23.6	-	-
**27**	Domestic canary	*Serinus canaria domestica*	swab	CnBV-2	neg	29.5	neg	23.2	-	-	-	-	20.7	-	-
**28**	Domestic canary	*Serinus canaria domestica*	swab	CnBV-2	neg	32.2	neg	24.3	-	-	-	-	21.4	-	-
**29**	Domestic canary	*Serinus canaria domestica*	p/c swab	CnBV-2	neg	34.7	neg	28.9	-	-	-	-	25.8	-	-
**30**	Domestic canary	*Serinus canaria domestica*	p/c swab	CnBV-2	neg	37.2	neg	33.2	-	-	-	-	30.1	-	-
**31**	Domestic canary	*Serinus canaria domestica*	p/c swab	CnBV-2	neg	35.8	neg	30.4	-	-	-	-	28.3	-	-
**32**	Domestic canary	*Serinus canaria domestica*	brain	CnBV-2	neg	18.5	neg	14.1	-	-	-	-	11.2	-	-
**33**	Domestic canary	*Serinus canaria domestica*	swab	CnBV-2	neg	32.4	neg	24.6	-	-	-	-	22.3	-	-
**34**	Domestic canary	*Serinus canaria domestica*	brain	CnBV-2	neg	19.8	neg	15.1	-	-	-	-	12.4	-	-
**35**	Domestic canary	*Serinus canaria domestica*	brain	CnBV-2	neg	22.1	neg	16.9	-	-	-	-	14.6	-	-
**36**	Domestic canary	*Serinus canaria domestica*	brain	CnBV-2	neg	21.5	neg	17.7	-	-	-	-	15.2	-	-
**37**	Domestic canary	*Serinus canaria domestica*	brain	CnBV-2	neg	18.6	neg	12.9	-	-	-	-	11.1	-	-
**38**	Domestic canary	*Serinus canaria domestica*	brain	CnBV-3	neg	16.8	neg	12.2	-	-	-	-	14.9	-	-
**39**	Domestic canary	*Serinus canaria domestica*	brain	CnBV-3	neg	21.6	neg	14.5	-	-	-	-	12.5	-	-
**40**	Yellow-winged pytilia	*Pytilia hypogrammica*	brain	EsBV-1	neg	18.0	neg	14.8	-	-	-	-	27.6	-	-
**41**	Black-rumped waxbill	*Estrilda troglodytes*	brain	EsBV-1	neg	20.3	neg	16.3	-	-	-	-	28.9	-	-
**42**	Black-faced firefinch	*Lagonosticta larvata*	gi	neg	neg	neg	neg	neg	-	-	-	-	neg	-	-
**43**	Star finch	*Bathilda ruficauda*	gi	neg	neg	neg	neg	neg	-	-	-	-	neg	-	-
**44**	Zebra finch	*Taeniopygia guttata*	gi/brain	neg	neg	neg	neg	neg	-	-	-	-	neg	-	-
**45**	Domestic canary	*Serinus canaria domestica*	brain	neg	neg	neg	neg	neg	-	-	-	-	neg	-	-

Part 2

No.	Common name	Scientific name	Sample	Virus[a]	multiBornaX			panBorna 7.2	PaBVcon MD	PaBVcon PG	PaBV-2 P	PaBV-4 P	CnBV-2 P	ABBV-1 M	ABBVcon P
**46**	Kea	*Nestor notabilis*	cc[c]	PaBV-1	20.4	neg	neg	13.2	13.4	19.2	24.3	15.0	-	-	-
**47**	Kea	*Nestor notabilis*	cc[c]	PaBV-1	18.9	neg	neg	12.1	13.5	18.0	22.8	13.9	-	-	-
**48**	Galah	*Eolophus roseicapilla*	pv	PaBV-2	26.6	neg	neg	19.7	17.1	17.3	19.3	neg	-	-	-
**49**	African grey parrot	*Psittacus erithacus*	brain	PaBV-2	26.4	neg	neg	20.0	18.2	16.6	18.8	neg	-	-	-
**50**	Salmon-crested cockatoo	Cacatua moluccensis	pv/gi	PaBV-2	35.4	neg	neg	21.2	18.2	18.5	20.2	neg	-	-	-
**51**	Sulphur-crested cockatoo	*Cacatua galerita*	swab	PaBV-2	31.7	neg	neg	24.9	22.7	20.9	22.4	neg	-	-	-
**52**	Cockatiel	*Nymphicus hollandicus*	organ pool	PaBV-2	21.4	neg	neg	14.4	12.5	11.2	13.6	neg	-	-	-
**53**	Sulphur-crested cockatoo	*Cacatua galerita*	swab	PaBV-4	38.9	neg	neg	35.8	27.4	30.2	33.0	27.4	-	-	-
**54**	Sulphur-crested cockatoo	*Cacatua galerita*	swab	PaBV-4	28.9	neg	neg	26.0	21.3	22.0	25.5	19.3	-	-	-
**55**	Galah	*Eolophus roseicapilla*	swab	PaBV-4	35.3	neg	neg	31.1	25.4	27.5	30.2	24.9	-	-	-
**56**	Red-tailed black cockatoo	*Calyptorhynchus banksii*	brain	PaBV-4	24.7	neg	neg	15.8	13.2	13.1	19.7	12.3	-	-	-
**57**	Gang-gang cockatoo	*Callocephalon fimbriatum*	skin/ lung	PaBV-4	32.1	neg	neg	22.2	15.3	20.4	24.3	15.7	-	-	-
**58**	Moluccan king parrot	*Alisterus amboinensis*	brain	PaBV-4	(40.3)	neg	neg	neg	27.8	33.9	neg	28.9	-	-	-
**59**	Blue-naped parrot	*Tangygnathus lucionensis*	brain	PaBV-4	24.2	neg	neg	20.3	18.1	20.2	23.9	16.2	-	-	-
**60**	Scarlet macaw	*Ara macao*	brain	PaBV-4	25.0	neg	neg	21.7	18.3	22.4	26.3	16.9	-	-	-
**61**	Red-shouldered macaw	*Diopsittaca cumanensis*	brain	PaBV-4	22.9	neg	neg	18.7	13.8	18.1	21.9	13.1	-	-	-
**62**	Scarlet-fronted parakeet	*Psittacara wagleri*	brain	PaBV-4	22.2	neg	neg	19.1	15.8	14.1	17.6	12.6	-	-	-
**63**	Austral parakeet	*Enicognathus ferrugineus*	brain	PaBV-4	32.2	neg	neg	27.8	23.4	22.7	26.1	21.1	-	-	-
**64**	Grey parrot	*Psittacus erithacus*	swab	PaBV-4	38.2	neg	neg	neg	33.9	33.5	35.3	30.7	-	-	-
**65**	Blue-throated macaw	*Ara glaucogularis*	organ pool	PaBV-4	29.5	neg	neg	20.7	16.7	15.9	20.1	14.1	-	-	-
**66**	Grey parrot	*Psittacus erithacus*	organ pool	PaBV-4	neg	neg	neg	31.1	26.2	26.3	29.4	23.7	-	-	-
**67**	Red-and-green macaw	*Ara chloroptera*	organ pool	PaBV-4	24.6	neg	neg	19.4	15.3	14.3	17.8	12.6	-	-	-
**68**	unknown psittacine	*Psittaciformes sp.*	brain	PaBV-4	19.7	neg	neg	16.9	12.5	12.3	15.9	10.9	-	-	-
**69**	Sun parakeet	*Aratinga solstitialis*	brain	PaBV-4	19.5	neg	neg	20.2	11.9	12.0	15.2	10.6	-	-	-
**70**	unknown psittacine	*Psittaciformes sp.*	organ pool	PaBV-4	(39.5)	neg	neg	27.0	19.3	19.9	23.2	18.1	-	-	-
**71**	unknown psittacine	*Psittaciformes sp.*	organ pool	PaBV-4	neg	neg	neg	neg	29.9	neg	neg	30.0	-	-	-
**72**	Sulphur-crested cockatoo	*Cacatua galerita*	swab	PaBV-4	neg	neg	neg	neg	neg	35.6	37.1	33.4	-	-	-
**73**	unknown psittacine	*Psittaciformes sp.*	blood	PaBV-4	(39.1)	neg	neg	neg	38.6	neg	37.9	33.1	-	-	-
**74**	Salmon-crested cockatoo	Cacatua moluccensis	brain	PaBV-4/7	24.5	neg	neg	16.1	13.7	19.1	23.1	17.1	-	-	-
**75**	Salmon-crested cockatoo	Cacatua moluccensis	pv	PaBV-4/7	25.6	neg	neg	17.1	14.4	22.8	27.4	21.0	-	-	-
**76**	Turquoise-fronted amazon	*Amazona aestiva*	swab	PaBV-7	30.3	neg	neg	23.2	21.0	31.6	32.9	neg	-	-	-
**77**	Blue-and-yellow macaw	*Ara ararauna*	swab	ns	neg	neg	neg	neg	neg	neg	neg	37.2	-	-	-
**78**	Major Mitchell's cockatoo	*Lophochroa leadbeateri*	gi	neg	neg	neg	neg	neg	neg	neg	neg	neg	-	-	-
**79**	Rosy-faced lovebird	*Agapornis roseicollis*	gi	neg	neg	neg	neg	neg	neg	neg	neg	neg	-	-	-
**80**	Dusky parrot	*Pionus fuscus*	gi/brain	neg	neg	neg	neg	neg	neg	neg	neg	neg	-	-	-
**81**	Cape parrot	*Poicephalus robustus*	gi	neg	neg	neg	neg	neg	neg	neg	neg	neg	-	-	-
**82**	African grey parrot	*Psittacus erithacus*	gi/brain	neg	neg	neg	neg	neg	neg	neg	neg	neg	-	-	-

[a] Viruses were identified by Sanger sequencing

[b] Simultaneous infection with PaBV-4 and PaBV-7

[c] Cell culture isolates were used as no original samples of PaBV-1-infected individuals were available.

Table S3. Primers used for amplification and cloning of X gene reference sequences.

Virus	Forward primer	Sequence 5' → 3'	Reverse primer	Sequence 5' → 3'
PaBV-1	P1_for	CAA TGT CTC CTG AGA TAG	P1_rev	GAT AGT GAT TCA AGC CCA G
PaBV-2	P2_for	CAA TGT CGC CCG AAA TAG	P2_rev	GAG AGT GAT TCG AGC CCA G
PaBV-4	P4-for	CAA TGT CCC CTG AGA TAG	P4_rev	GAG AGT GAT TCA AGT CCT G
PaBV-7	P7_for	CGA TGT CTC CGG AGA TTG	P7_rev	GAC AAT GAC TCA AGA CCA G
CnBV-1	C1_for	CTT TCA GAC GAG ATT GCA G	C1_rev	GAT AAT GAC TCA AAT CCC AG
CnBV-2	C2_for	CTT TCA GAA GAG ATA GCC	C2_rev	GAG AGA GAT TCA AAT CCA G
CnBV-3	C3_for	CTT TCA GCC GAG ATA GCT G	C3_rev	GAG AGT GAT TCA AAT CCT GAG
EsBV-1	E1_for	CCT TTC AGA TGA AAT TGC	E1_rev	GAC AGT GAT TCA AGG CCT G
ABBV-1	A1_for	CCT TTC TGA TGA AAT TGC	A1_rev	GAT AAA GAT TCA AAA CCA G
BoDV-1	B_for	GGG CAG AGC TCT CAG G	B_rev	GAG GGC GGA CAG GGA C
BoDV-2	B2A_for	GAT GGG GCA GAG CTC TCA GG	B2A_rev	CCT GGA GTG CTG ACA GGG AC

Table S4. Primers and probes for conventional and rRT-PCR assays for the detection of orthobornaviruses.

Assay	Primer/probe name	Sequence (5' to 3')	Final concentration (µM)	Reference
Ncon	BornaNconF	CCHCATGAGGCTATWGATTGGATTAACG	0.2	[4]
	BornaNconR	GCMCGGTAGCCNGCCATTGTDGG	0.2	
Mcon	BornaMconF	GGRCAAGGTAATYGTYCCTGGATGGCC	0.2	[4]
	BornaMconR	CAACACCAATGTTCCGAAGMCG	0.2	
Mcon-W	Borna_M_Forward	CAA GGT AAT YGT YCC TGG ATG G	0.4	[24]
	Borna_M_Reverse	ACC AAT GTT CCG AAG MCG AWA Y	0.4	
panBorna_7.2	panBorna_1319+	CGCGACCMTCGAGYCTRGT	0.8	[28]
	panBorna_1529-	GACARCTGYTCCCTTCCKGT	0.8	
	panBorna_1471.2_P	FAM-AAGAAYCCHTCCATGATCTCMGAYCMAGA-BHQ1	0.4	
PaBVcon_MD	PaBVcon_1908+	CAAGGTAATYGTYCCTGGATGGCC	0.8	[10]
	PaBVcon_2008-	TCACTGAAAGAAANGGTATRTTGAT	0.8	
	PaBVcon_1937_P	FAM-TAATGTTGGARATAGACTTTGTTGG-BHQ1	0.2	
PaBVcon_PG	PaBVcon_1401+	AAGAAGAAYCCYTCCATGATCTC	0.8	[29]
	PaBVcon_1483-	AAYTGCCGAATBARGTCATC	0.8	
	PaBVcon_1461as_P*	FAM-TCGATAACTGYTCCCTTCCGGTC-BHQ1	0.2	
PaBV-4_P	PaBV-4_1305+	CAGACAGCACGTCGAGTGAGA	0.8	[3]
	PaBV-4_1370-	AGTTAGGGCCTCCCTGGGTAT	0.8	
	PaBV-4_1329_P	FAM-AGGTCCCCGCGAAGGAAGCGA-BHQ1	0.2	
PaBV-2_P	PaBVcon_1401+	AAGAAGAAYCCYTCCATGATCTC	0.8	[23,29]
	PaBV-2_1482-	ATTGCCGAATCAGGTCATCA	0.8	
	PaBVcon_1461as_P	FAM-TCGAYAACTGYTCCCTTCCGGTC-BHQ1	0.2	
CnBV-2_P	CnBV-2_1392+	CCAGCCGGTAGAGCATCTTC	0.8	[22]
	CnBV-2_1484-	TTCGACAACTGCTCCCTTCC	0.8	
	CnBV-2_1430_P	FAM-ACCCATCCATGATCTCCGACCCAGACC-BHQ1	0.2	
ABBV-1_M	ABBV-1_2052+	CGAGGGAGAAGARACTGGTTGATT	0.8	this study
	ABBV-1_2172-	ACYGCCAAAGAGTTRAGYGT	0.8	
	ABBV-1_2111_P	FAM-ATGTGGAACCYGCTGGTCACTCA-BHQ1	0.2	
ABBVcon_P	ABBVcon_1407+	TGCYCTGACACAACCAGTCGA	0.8	this study
	ABBVcon_1510-	CATTCGACAATTGCTCCCTTCC	0.8	
	ABBVcon_1459_P	FAM-TCCATGATCTCAGACCCMGACCA-BHQ1	0.2	

Table S5. Impact of the presence of multiple avian bornavirus RNAs on the rRT-PCR performance. Multiple *in vitro*-transcribed control RNAs (10^6 copies per reaction of each virus) used as templates for the multiplex rRT-PCR.

	**BornaP_Fam** C_T	**BornaC_Aby** C_T	**BornaA_A674N** C_T
**PaBV-1/ CnBV-1/ ABBV-1**	23.95	26.92	24.71
**PaBV-1/ CnBV-2/ ABBV-1**	23.89	24.22	24.65
**PaBV-1/ CnBV-3/ ABBV-1**	24.23	26.25	24.94
**PaBV-1/ EsBV-1/ ABBV-1**	24.09	24.96	25.02
**PaBV-2/ CnBV-1/ ABBV-1**	29.26	27.03	24.59
**PaBV-2/ CnBV-2/ ABBV-1**	29.08	24.26	24.31
**PaBV-2/ CnBV-3/ ABBV-1**	29.24	26.30	24.40
**PaBV-2/ EsBV-1/ ABBV-1**	29.85	25.41	24.85
**PaBV-4/ CnBV-1/ ABBV-1**	25.44	29.18	22.88
**PaBV-4/ CnBV-2/ ABBV-1**	25.06	22.62	22.24
**PaBV-4/ CnBV-3/ ABBV-1**	25.38	24.62	22.95
**PaBV-4/ EsBV-1/ ABBV-1**	25.17	23.66	22.67
**PaBV-7/ CnBV-1/ ABBV-1**	21.92	29.61	22.46
**PaBV-7/ CnBV-2/ ABBV-1**	22.16	22.98	22.90
**PaBV-7/ CnBV-3/ ABBV-1**	22.13	24.78	22.98
**PaBV-7/ EsBV-1/ ABBV-1**	21.74	23.78	22.91
